# Absence of Role of Dietary Protein Sensing in the Metabolic Benefits of Duodenal-Jejunal Bypass in the Mouse

**DOI:** 10.1038/srep44856

**Published:** 2017-03-23

**Authors:** Aude Barataud, Daisy Goncalves, Jennifer Vinera, Carine Zitoun, Adeline Duchampt, Amandine Gautier-Stein, Gilles Mithieux

**Affiliations:** 1 Institut National de la Santé et de la Recherche Médicale, U1213, Lyon, F-69008, France; 2 Université de Lyon, Lyon, F-69008, France; 3 Université Lyon 1, Villeurbanne, F-69622, France

## Abstract

Roux-en-Y gastric bypass (RYGB) induces remission or substantial improvement of type 2 diabetes mellitus (T2D) but underlying mechanisms are still unclear. The beneficial effects of dietary proteins on energy and glucose homeostasis are mediated by the antagonist effects of peptides toward mu-opioid receptors (MORs), which are highly expressed in the distal gut. We hypothesized that the beneficial effects of RYGB could depend at least in part on the interaction of peptides from food with intestinal MORs. Duodenal-jejunal bypass (DJB) was performed in obese and lean wild-type (WT) or MOR deficient (MOR^−/−^) mice. Food intake and body weight was monitored daily during 3 weeks. Glucose homeostasis was assessed from glucose and insulin tolerance tests. In obese WT and MOR^−/−^ mice, DJB induced a rapid and sustained weight loss partly independent of food intake, and a rapid improvement in glycaemic parameters. Weight loss was a major determinant of the improvements observed. In lean WT and MOR^−/−^ mice, DJB had no effect on weight loss but significantly enhanced glucose tolerance. We found that MORs are not essential in the metabolic beneficial effects of DJB, suggesting that protein sensing in the distal gut is not a link in the metabolic benefits of gastric surgery.

Bariatric surgeries have emerged as the most effective and durable therapies to treat obesity and one of its comorbidities, type 2 diabetes mellitus (T2DM)^1^,^2^. The Roux-en-Y gastric bypass (RYGB) procedure is one of the most performed and most efficient bariatric surgery. This surgery involves the creation of a small gastric pouch directly connected with the mid-jejunum and the diversion of the biliopancreatic secretions into the distal jejunum. After RYGB surgery, patients exhibit a calorie intake reduction and a considerable and lasting weight loss of up to 30%^2^,^3^. Improvement or remission of T2DM is observed in approximately 80% of patients^4^,^5^, but surprisingly the improvements in glycaemic control cannot be correlated exclusively to the extent of weight loss. Indeed, many type 2 diabetic patients stop their medication within days after surgery, before any significant body weight loss^6^. Thus, understanding the mechanisms underlying the metabolic improvements initiated by RYGB could lead to the development of less invasive treatments of T2DM.

A particularity of RYGB is that the gut anatomy is changed, which results in a modification in nutrients routing. It is well known that macronutrients act as signalling molecules and, in particular, proteins are known to induce beneficial effects on health. Protein-enriched diets have proven their efficacy in promoting satiety^7^,^8^ and in maintaining weight loss^9^,^10^,^11^. Moreover, several studies have shown that protein-enriched diets, ingested over a short or a long period of time, decrease postprandial blood glucose and improve overall glucose control in patients with T2DM^12^,^13^. Therefore, the question arises as to whether dietary proteins could play a role in the metabolic improvements induced by RYGB.

Recently, we demonstrated that mu-opioid receptors (MORs) are essential in mediating the satiety effects of dietary proteins^14^. We found that infusions of various peptides directly into the portal vein of rodents inhibit the MORs surrounding the portal vein. This inhibition leads to satiety via the activation of a gut-brain neural circuitry implicating intestinal gluconeogenesis. The causal role of MORs was confirmed by the insensitivity of MOR-knockout (MOR^−/−^) mice to the satiety effects induced by protein-enriched diet^14^. Remarkably, MORs are highly expressed in the distal gut^15^,^16^,^17^ where food is directly diverted after RYGB. So, it can be assumed that after RYGB peptides from food might have direct access to the MORs in the distal gut, besides the MORs surrounding the portal vein. Our hypothesis was this could amplify the peptide effects and constitute an explanation of the dramatic metabolic benefits of RYGB.

In this study, we assessed whether MORs are involved in the short-term metabolic effects of RYGB, either in a context of obesity or of leanness. Since we were primarily interested in the role of gut remodelling in the beneficial effects of RYGB, we performed a duodenal-jejunal bypass (DJB) surgery, which is a simplified RYGB that does not imply any gastric volume restriction (Fig. 1), in both wild-type and MOR^−/−^ mice.

## Materials and Methods

### Study approval

All the experiments involved in this study were carried out in accordance with the principles and guidelines established by the European Convention for the Protection of Laboratory Animals and approved by our regional animal care committee (C2EA-55, Université Lyon 1, Lyon).

### Mice and diets

Male C57Bl/6 J wild-type mice (WT) were purchased from Charles River Laboratories at 4 weeks of age. Mu-opioid receptors knock-out mice (MOR^−/−^) were generated in our facilities from two couples of mice obtained from The Jackson Laboratory (Oprm1^tm1Kff^/J)^18^. All mice were housed in the animal facility of Lyon 1 University under controlled temperature (22 ± 2 °C) and lighting (12 h light/dark cycle with light at 7 a.m.) with free access to food and water. Before surgery, mice were group-housed (2–4 mice per cage) and after surgery mice were individually housed to allow better healing and improve recovery.

In order to induce obesity, 4-weeks old WT and MOR^−/−^ mice were given ad libitum a high-fat/high-sucrose (HF-HS) diet during 20 weeks prior to surgery. HF-HS diet, consisting of 36.1% fat (butter and soybean oil (14:1)), 35% carbohydrates (50% maltodextrine +50% sucrose) and 19.8% proteins (casein from bovine milk), was produced by the Unité de Préparation des Aliments Expérimentaux (INRA, UE0300, Jouy-en-Josas, France). For studies in lean animals, WT and MOR^−/−^ mice were maintained on standard diet (SAFE A04, Augis, France) and surgery was performed at 14-weeks old. All the animals were maintained on their respective diet after surgery.

### Surgical Procedures

#### Surgical preparation

Food and water were not restricted before surgery. Anaesthesia was induced and maintained throughout the operation with Isoflurane and air/oxygen. During anaesthesia, mice were placed on a temperature-controlled heating pad to avoid hypothermia. Ophthalmic ointment (Ophtalon^®^) was applied on mice eyes to prevent drying and the abdomen was shaved and disinfected. During the entire operation, organs exposed to air were kept moist by application of NaCl 0.9%.

#### DJB surgery

**Figure 1:**
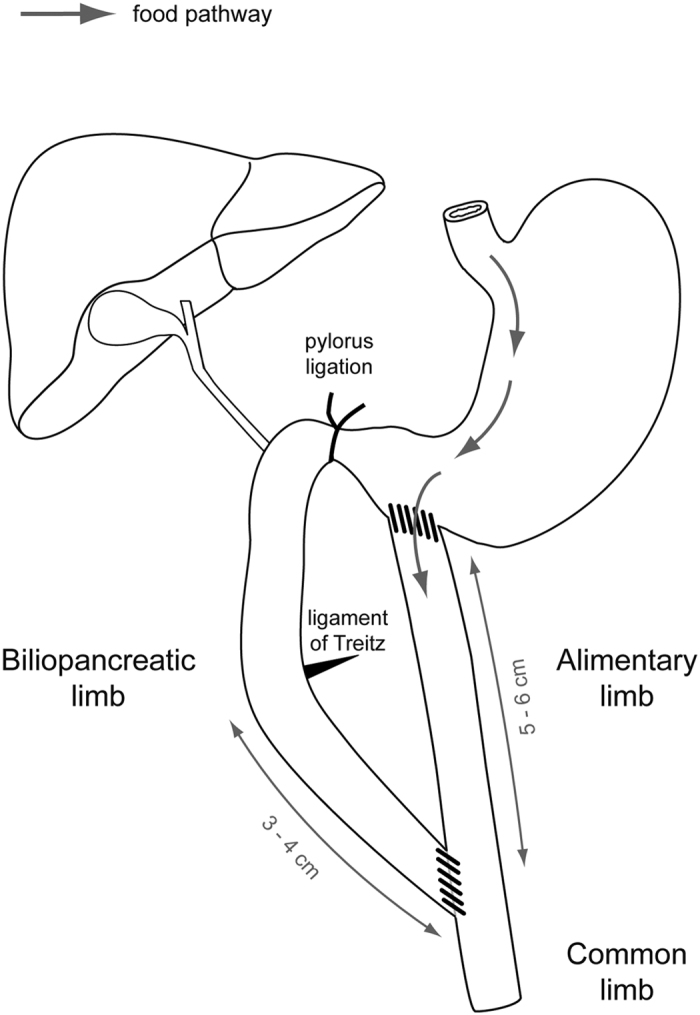
Schematic representation of the duodenal-jejunal bypass in the mouse. Jejunum was transected 3–4 cm downstream of the ligament of Treitz and the proximal jejunum was connected to the intestine 5–6 cm beyond the transversal section. Then distal jejunum was anastomosed to the stomach and finally pylorus was ligated. This procedure allows the creation of a biliopancreatic limb and an alimentary limb of about 5–6 cm each and a common limb of approximately 18–20 cm long.

A 3 cm midline laparotomy was performed and small bowel was exposed to the surgeon. To achieve the duodenal-jejunal bypass as presented in Fig. 1, the first step of the procedure consisted in creating the biliopancreatic limb. To this end, the ligament of Treitz was identified and jejunum was transected 3–4 cm distal to this ligament. Then, a longitudinal and antimesenteric incision (approximately 0.5 cm length) was performed 5–6 cm distal to the transected bowel. Subsequently, the proximal jejunum was moved close to the longitudinal incision of the distal jejunum and the two parts of the gut were anastomosed in an end-to-side fashion using a continuous suture (Ethicon nylon 8-0). The second step of the surgery was the building of the alimentary limb. For this purpose, the stomach was exposed and an incision of 0.5 cm length was performed near to the pylorus, in a poor vascularized portion of the stomach. Then an end-to-side gastrojejunostomy was created with a 8-0 nylon running suture (Ethicon). The third step of the procedure involved the ligation of the pylorus in order to avoid the passage of food to the biliopancreatic limb. Therefore, the pylorus was isolated and surrounded by a 6-0 nylon suture thread (Ethicon) and three simple knots were made. Throughout the DJB surgery, a particular attention was given to stop potential bleeding. For this reason hemostatic compresses (Pangen) were applied to the intestine and stomach after incisions and sutures were started only after the complete cessation of bleeding. After gently returning the small intestine and stomach inside the abdominal cavity, the abdominal wall and the skin were closed using 6-0 nylon running and interrupted suture respectively (Ethicon).

#### Sham surgery

The sham operation consisted of a 3-cm midline laparotomy. Sham-operated and sham-operated pair-fed mice (sham-PF) underwent the same duration of anaesthesia as DJB-treated mice (1h15) and the abdominal wall and skin were closed using running and interrupted suture respectively (Ethicon 6-0 nylon). The sham-PF mice were pair-fed to match the daily food intake of the DJB mice.

#### Postoperative care

ketoprofen (5 mg/kg) was administered by an intraperitoneal injection before the abdominal wall closure and 1.5 mL of NaCl 0.9% was injected subcutaneously at the end of the surgery. Postoperative analgesia consisted in a daily injection of ketoprofen (5 mg/kg, s.c.) for 4 days after surgery. Mice were housed in individual cages and put on heating pad set at 37 °C during 5 days. After the surgery, mice were given ad libitum access to water but were deprived of food during 24 h. In the postoperative day 1, mice were given approximately 2 mL of liquid diet (Ensure). From the second postoperative day, mice were fed ad libitum with the appropriate food (HF-HS or standard diet), except for sham-PF mice.

### Body Weight and Food Intake

Body weight and food intake was monitored daily during 3 weeks. HF-HS diet was changed 3 times a week for ad libitum fed mice (sham-operated and DJB-treated mice) and daily for sham-PF mice.

### Glucose and Insulin Tolerance Tests

Glucose tolerance test (GTT) and insulin tolerance test (ITT) were carried out 2 and 3 weeks after the surgery, respectively. Animals were fasted for 16 (GTT) or 6 hrs (ITT) and then received an intraperitoneal injection of glucose (1 g/kg) or insulin (0.75 IU/kg for obese mice and 0.5 IU/kg for lean mice, Insulatard, Novo Nordisk). Blood glucose was monitored for 120 (GTT) or 90 min (ITT) using a glucometer (Accu-Check, Roche) From samples collected from the tip of the tail vein. Insulinemia was quantified using an ELISA kit (Mercodia).

### Statistical analysis

The results were expressed as mean ± SEM. Unpaired Student’s t test was used for two-group comparisons. One-way ANOVA followed by Tukey’s post-hoc test was used for three-group comparisons. p < 0.05 was considered significant.

## Results

### Duodenal-jejunal bypass induces weight loss and metabolic improvements in obese wild-type mice

**Figure 2:**
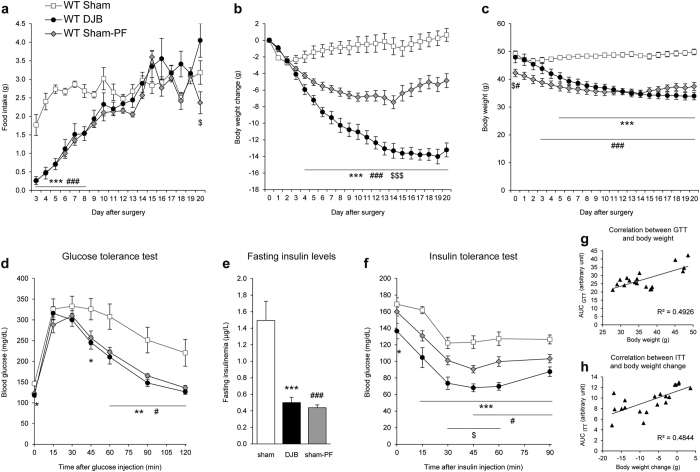
Effects of duodenal-jejunal bypass on food intake, body weight and glucose homeostasis in wild-type obese mice. (**a**) Evolution of food intake, (**b**) body weight change and (**c**) body weight of WT obese mice fed a HF-HS diet after DJB (black circles), sham (white squares) or sham pair-fed surgery (grey diamonds). (**d**) Glucose tolerance test was performed 2 weeks after surgery, (**e**) blood insulin levels were determined in 16-hour fasting mice and (**f**) insulin tolerance test was performed 3 weeks after the surgery. (**g**) Scatter plot of the values of the area under the curve of GTT vs weight for each mice and (**h**) scatter plot of the values of the area under the curve of ITT vs weight loss for each mice. The linear regression is annotated with Pearson’s correlation coefficient (r). n = 8 for WT DJB group, n = 6 for WT sham and WT sham-PF groups; *p < 0.05, **p < 0.01 and ***p < 0.001 for DJB vs sham group; ^#^p < 0.05 and ^###^p < 0.001 for sham-PF vs sham group; ^$^p < 0.05 and ^$$$^p < 0.001 for DJB vs sham-PF group.

We first studied the metabolic effects of DJB in obese WT mice fed a high-fat/high-sucrose (HF-HS) diet. Food intake after DJB was transiently reduced from postoperative day 3 to day 8 compared with WT sham group (Fig. 2a). Beyond the 8^th^ day post-surgery, WT DJB mice stabilized their food intake and reached the same level as that of WT sham mice. These results indicated that DJB did not induce a long lasting decrease in food intake. However, DJB in WT obese mice led to a significant and sustained weight loss compared to sham surgery. Indeed, WT DJB mice showed a steeply weight loss for the first 15 days (−28%, i.e. −13.7 g, Fig. 2b) and stabilized their body weight around 34 g (Fig. 2c) until the end of the study. To distinguish the effects specific of DJB surgery from those related to the decrease in food intake, a group of sham-operated mice pair-fed with DJB mice was studied (WT sham-PF, grey diamond, Fig. 2). As expected, sham-PF mice lost weight but to a lesser extent than DJB mice (maximal weight change: −7.5 ± 0.7 g at 14 days post-surgery, Fig. 2b) and tended to exhibit a gradual weight regain from the second week after the surgery. Thus, DJB in WT obese mice induced a significant weight loss partially independent of food intake.

Then we evaluated the effects of DJB on glycaemic control. Analysis of glucose tolerance test data revealed that DJB induced a significant improvement in glucose tolerance associated with a decrease in basal glycaemia (Fig. 2d) and a 3 fold decrease in blood insulin levels (Fig. 2e) compared to sham WT mice. Similar improvements in glycaemic control were observed in sham PF mice (Fig. 2d and e). It is noteworthy that the area under the curve of glycaemia during GTT was positively correlated with body weight (Fig. 2g). Relating to insulin sensitivity, WT DJB mice exhibited a substantial reduction in their blood glucose levels after insulin injection compared with those of sham WT mice (Fig. 2f). Sham-PF WT mice displayed a smaller but significant enhancement in their insulin tolerance compared with sham WT mice, with no improvement in fasting glycaemia (Fig. 2f). Interestingly, the area under the curve of glycaemia during ITT was positively correlated with body weight loss (Fig. 2h). Together, these data suggest that the improvements in glucose and insulin tolerance after DJB in obese WT mice are mainly associated with weight loss.

### Duodenal-jejunal bypass induces weight loss and metabolic improvements in obese MOR^−/−^ mice

**Figure 3:**
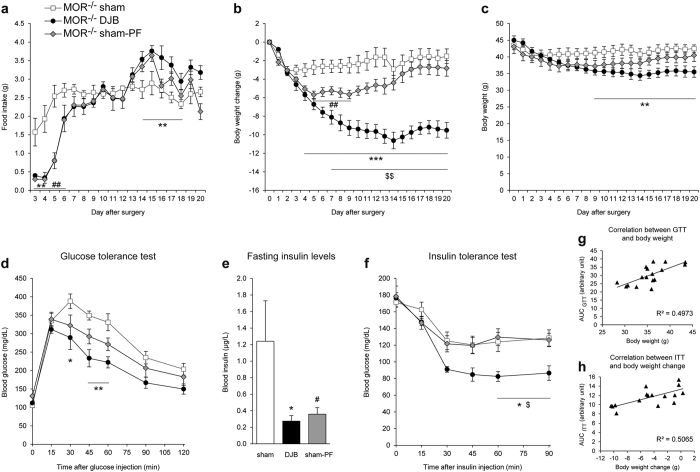
Effects of duodenal-jejunal bypass on food intake, body weight and glucose homeostasis in MOR^−/−^ obese mice. (**a**) Evolution of food intake, (**b**) body weight change and (**c**) body weight of MOR^−/−^ obese mice fed a HF-HS diet after DJB (black circles), sham (white squares) or sham pair-fed surgery (grey diamonds). (**d**) Glucose tolerance test was performed 2 weeks after surgery, (**e**) blood insulin levels were determined in 16-hour fasting mice and (**f**) insulin tolerance test was performed 3 weeks after the surgery. (**g**) Scatter plot of the values of the area under the curve of GTT vs weight for each mice and (**h**) scatter plot of the values of the area under the curve of ITT vs weight loss for each mice. The linear regression is annotated with Pearson’s correlation coefficient (r). n = 5 for MOR^−/−^ DJB group, n = 6 for MOR^−/−^ sham and MOR^−/−^ sham-PF groups; *p < 0.05, **p < 0.01 and ***p < 0.001 for DJB vs sham group; ^#^p < 0.05 and ^##^p < 0.01 for sham-PF vs sham group; ^$^p < 0.05 and ^$$^p < 0.01 for DJB vs sham-PF.

To assess the involvement of MORs in the metabolic improvements observed after DJB, we performed the same experiments in MOR^−/−^ obese mice. As in WT mice, DJB in MOR^−/−^ obese mice led to an initial hypophagia from postsurgical day 3 to day 6 and then to a stabilization in their food intake at the same level as MOR^−/−^ sham mice (around 2.5 g/day, Fig. 3a). As in WT mice, DJB induced a decrease in body weight in MOR^−/−^ mice, independently of food intake. Indeed, MOR^−/−^ DJB mice gradually lost weight during 14 days (−24% i.e. −10.6 g, Fig. 3b) and then stabilized their body weight around 35 g (Fig. 3c), whereas PF-MOR^−/−^ lost weight during the first week (maximal weight loss: −5.7 ± 0.4 g on day 5 post-surgery) and regained weight from the 9^th^ post-surgical day until the end of the experiment (+2.8 g between day 9 and 20, Fig. 3b). Taken together, these data strongly suggested that DJB induced almost identical effects on food intake and body weight in WT and MOR^−/−^ obese mice.

DJB performed in MOR^−/−^ mice induced an improvement in glucose tolerance (Fig. 3d). MOR^−/−^ sham-PF mice showed a trend toward an improvement in glucose tolerance but the results were not statistically significant (Fig. 3d). In addition, DJB and pair feeding induced a marked decrease in blood insulin basal levels (Fig. 3e). As in WT mice, these improvements were positively correlated with body weight (Fig. 3g). MOR^−/−^ DJB mice exhibited improved insulin tolerance compared with sham MOR^−/−^ mice (Fig. 3f), whereas MOR^−/−^ sham-PF mice did not display improvement in insulin tolerance compared with sham MOR^−/−^ mice. However, as for WT mice, the improvement in insulin tolerance induced by DJB was positively correlated with body weight loss (Fig. 3h). Taken together, these data suggest that as in WT mice, the improvements in glucose and insulin tolerance after DJB in obese MOR^−/−^ mice are mainly associated with weight loss.

### Duodenal-jejunal bypass does induce neither food intake decrease nor weight loss in lean wild type and MOR^−/−^ mice

Since the decrease in body weight had a major impact on the metabolic improvements induced by DJB, we studied the metabolic effects of this surgery in lean mice. Indeed, data from the literature reported that in lean mice bariatric surgery caused little or no weight loss^19^,^20^.

**Figure 4:**
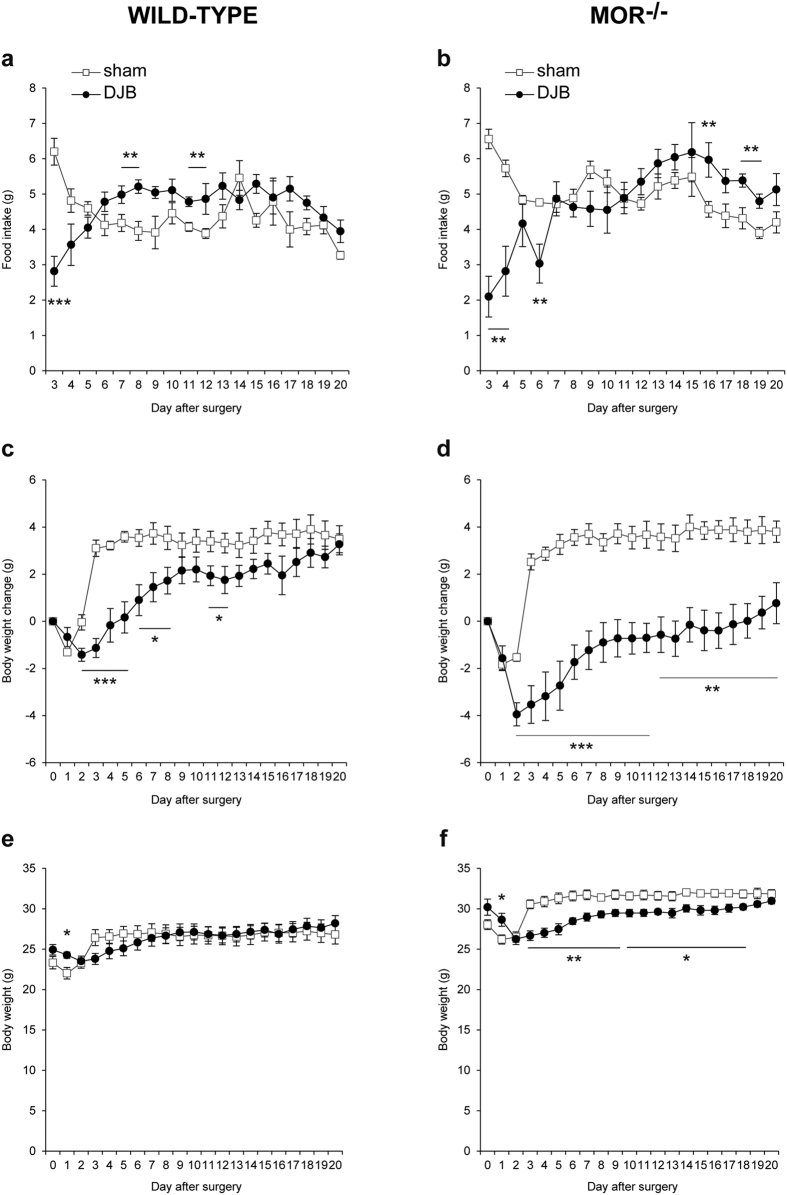
Effects of duodenal-jejunal bypass on food intake and body weight in wild-type and MOR^−/−^ lean mice. (**a**,**b**) Evolution of food intake, (**c**,**d)** body weight change and (**e**,**f**) body weight of WT and MOR^−/−^ lean mice fed a standard diet after DJB (black circles) or sham surgery (white squares). n = 6 for WT DJB group, n = 7 for WT sham group, n = 7 for MOR^−/−^ DJB group, n = 7 for MOR^−/−^ sham group; *p < 0.05, **p < 0.01 and ***p < 0.001 vs sham group.

DJB and sham surgeries were performed in 14-weeks old WT and MOR^−/−^ mice fed a standard diet. As illustrated in Fig. 4a, DJB in WT lean mice resulted in an early short phase of hypophagia (from the first day to postoperative day 3) followed and counterbalanced by a short period of hyperphagia (during the second postsurgical week), then food intake resumed initial basal level. In MOR^−/−^ mice, DJB induced comparable outcomes except that MOR^−/−^ DJB mice displayed a longer hypophagia period and a slightly delayed hyperphagia rebound (Fig. 4b). Thus, DJB performed in WT or MOR^−/−^ mice did not induce a lasting decrease in food intake.

Wild type DJB and sham mice exhibited a similar body weight from the second day after surgery (Fig. 4e). When DJB mice were refed ad libitum (i.e., from postsurgical day 2), they regained weight (Fig. 4c). However, this weight regain occurred more slowly in WT DJB mice compared with sham WT mice. This can be explained by the short initial hypophagia experienced by the DJB mice (Fig. 4c). Since MOR^−/−^ DJB mice displayed a slightly longer hypophagia period compared with WT DJB mice, they also regained weight more slowly (Fig. 4d) and completely resumed their initial weight only 14 days after the surgery (Fig. 4f). Therefore, MOR^−/−^ DJB mice displayed a statistically significant reduced body weight compared to MOR^−/−^ sham mice from postoperative day 4 to 18 (Fig. 4f), but it is to be noted that the weight difference between the 2 groups of mice was weak (less than 2.2 g on the second and third week after surgery), and there was no difference in body weight at the end of the study, when tolerance tests were performed (Fig. 4e and f). Together, these results highlighted that DJB does not induce lasting weight loss in either lean WT or lean MOR^−/−^ mice.

### Glucose tolerance is enhanced after duodenal-jejunal bypass in lean wild-type and MOR^−/−^ mice

To determine whether DJB affects glucose homeostasis in mice independently of weight loss, we carried out intraperitoneal glucose and insulin tolerance tests in operated lean mice.

**Figure 5:**
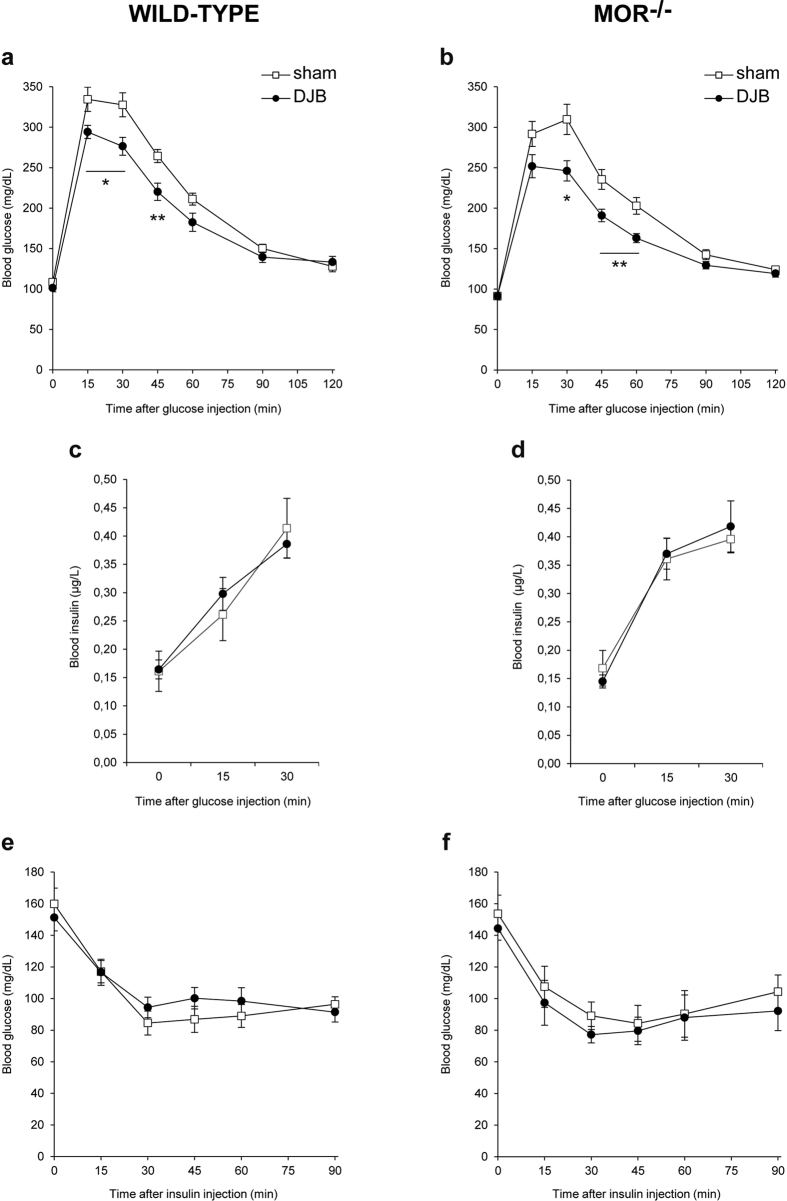
Effects of duodenal-jejunal bypass on glucose homeostasis in wild-type and MOR^−/−^ lean mice. (**a**,**b)** Glucose tolerance test was performed 2 weeks after surgery, (**c**,**d**) blood insulin levels were determined during the glucose tolerance test and (**e,f**) insulin tolerance test was performed 3 weeks after surgery in WT and MOR^−/−^ lean mice fed a standard diet after DJB (black circles) or sham surgery (white squares). n = 6 for WT DJB group, n = 7 for WT sham group, n = 7 for MOR^−/−^ DJB group, n = 7 for MOR^−/−^ sham group; *p < 0.05 and **p < 0.01 vs sham group.

Blood glucose levels were significantly reduced in WT DJB mice compared with WT sham mice 15, 30 and 45 min after glucose injection (Fig. 5a). MOR^−/−^ mice also displayed an improved glucose tolerance after DJB surgery compared with sham surgery (Fig. 5b). This glucose tolerance enhancement after DJB was not associated with any increase in blood insulin levels during the glucose tolerance test in WT and MOR^−/−^ mice (Fig. 5c and d, respectively).

Insulin tolerance data demonstrated that neither WT DJB mice nor MOR^−/−^ DJB mice exhibited improvement in insulin sensitivity compared with their counterpart sham mice (Fig. 5e for WT mice, Fig. 5f for MOR^−/−^ mice). Together, these data indicated that DJB performed in lean WT and MOR^−/−^ mice induces beneficial effects on glucose tolerance that are specific of surgery and independent of weight loss.

## Discussion

This paper addresses the question of the effects of DJB on glucose and energy homeostasis in obese and diabetic mice fed a high-calorie diet. We highlight that DJB in the mouse is a suitable model to analyse the metabolic consequences of the rearrangement of the intestinal tract produced by RYGB surgery. Indeed, obese mice undergoing DJB surgery exhibited a 30% weight loss and significant improvements in glucose tolerance and insulin sensitivity as observed after RYGB in humans. However, in this mouse model of DJB, the weight loss occurred within 2 weeks after the surgery, while in humans the same weight loss was observed only 6–12 months after RYGB. This substantial and early weight loss is nonetheless consistent with the findings of others that performed either RYGB^21^,^22^,^23^,^24^ or DJB in obese mice^25^, suggesting that gastric restriction is not necessary for weight loss after bariatric surgery in mice. Another result diverging from RYGB in humans is that DJB in mice does not induce a lasting reduction in calorie intake. In obese mice fed a HF-HS diet, DJB induced a transient decrease in food intake during the first postsurgical days. This may account for the recovery period after this heavy surgery. Then, DJB mice exhibited the same food intake as sham mice. This phenomenon was also observed in the mouse models of RYGB fed a high calorie diet, regardless of the gastric pouch size^21^,^24^,^25^. This absence of long-term decrease in calorie intake might be explained by the lack of gastric restriction associated with DJB surgery in rodents, which is not the case in patients undergoing RYGB, in which the gastric size is reduced. In addition, modifications in food preference were reported after the surgery to the benefit of healthy low-calorie diet intake^26^,^27^. Using sham-pair-fed mice, we finally observed that DJB in obese mice induces a substantial and sustained weight loss, which cannot be explained only by the decrease in food intake. This strongly suggests that additional benefits take place, e.g. increased energy expenditure and/or decreased energy absorption. An attractive possibility would be a change in the amount of specific intestinal bacteria conferring metabolic benefits as *Prevotella copri*^28^,^29^ or *Akkermansia muciniphila*^30^. It is noteworthy that *Akkermansia muciniphila* is increased after gastric bypass in morbidly obese patients^31^,^32^, and that a protein from its membrane is beneficial for glucose control by itself^33^.

Regarding the consequences of DJB on glucose homeostasis in obese and diabetic mice, our results suggest that the significant improvement in glucose tolerance was directly correlated to body weight. Moreover, insulin tolerance improved in proportion to weight loss. Adiposity was not assessed here. However, the skeletal muscle greatly contributes to glucose control. This warrants why body weight loss monitoring was preferred to adiposity monitoring in this study. The results obtained highlight that the early and substantial weight loss occurring after DJB might be sufficient *per se* to confer the improvements in glucose metabolism, confirming the studies with mice that were body-weight matched with RYGB mice^24^,^34^,^35^. Hence, DJB in obese mice could help in understanding the mechanisms underlying the rapid weight loss induced by bariatric surgeries.

Using this mouse DJB model, we studied the possible role of dietary protein in the beneficial effect of DJB. Since MORs are a key mechanistic link in the favourable effects induced by protein-enriched diets^14^, we used MOR^−/−^ mice to address this question. We first noted that these mice displayed a lower body weight than WT mice when they were fed a HF-HS diet. These results are consistent with data from the literature that revealed that MOR^−/−^ mice consuming a high-calorie diet gain less body weight than WT mice because of a lesser fat storage^36^,^37^. Nevertheless, DJB induced a rapid and massive weight loss in MOR^−/−^ obese mice, as in WT obese mice. Moreover, the positive correlations between glucose homeostasis and weight and between insulin sensitivity and weight loss induced by DJB were observed in MOR^−/−^ mice. This suggests that MORs are not implicated in the glycaemic parameters improvements in DJB. However, the possible role of MORs might be hidden by the benefits deriving from the dramatic weight loss in obese mice.

To question the weight loss-independent effects of DJB, we further performed this surgery in lean mice, as it has been reported that DJB induces little or no weight loss in the lean state^19^,^20^,^38^. According to our findings, DJB in WT lean mice improved glucose tolerance independently of weight loss as soon as 2 weeks after the surgery, whereas insulin tolerance was not modified post-surgery. These differences between lean and obese mice in the control of glucose homeostasis after DJB suggest that this surgery might induce its beneficial effects by targeting different metabolic processes depending on the initial host metabolism. It is accepted that GTT mobilizes physiological plasma concentrations of endogenous insulin, which alters EGP but not peripheral glucose uptake^39^. On the contrary, ITT involves supra-physiological plasma concentrations of exogenous insulin, which alters both EGP and peripheral glucose utilization^39^. Relating to lean animals in the absence of weight loss, it might thus be proposed that DJB improves insulin sensitivity of EGP, while not altering glucose utilization (Fig. 5). In contrast, in obese animals after weight loss the insulin sensitivity of both EGP and peripheral glucose utilization might improve (Figs 2 and 3). Regarding the potential role of MORs, we observed in lean mice that DJB surgery elicited almost identical effects on food intake, body weight change, glucose tolerance and insulin sensitivity in both MOR^−/−^ and WT mice. However, it is noteworthy that the effect of DJB on body weight in lean MOR^−/−^ mice was more pronounced than in lean WT mice (Fig. 4). These data are in keeping with a possible role of MOR in the modulation of body weight independently of food intake, as previously suggested^36^,^37^.

Therefore, the beneficial effects of bariatric surgery seem independent of MORs and/or of changes in the intestinal availability of dietary protein. This is consistent with the study of Swensson *et al*.^40^ conducted in patients undergoing gastric bypass procedure, which concluded that substituting a high-protein diet instead of the recommended standard low-fat diet after the surgery did not induce additional body weight loss. However, the consumption of a high-protein diet after weight loss induced by bariatric surgery appeared to be critical in preventing weight regain^41^ and maintaining free-fat mass^42^. Thus, further studies are needed to define precisely the role of dietary proteins supplementation in the beneficial effects of bariatric surgery. The use of a suitable mouse model of bariatric surgery subjected to different diets may help to address this issue.

In conclusion, this study strongly suggests: (1) DJB in obese mice promotes a sustained weight loss, which cannot be explained by the only decrease in food intake; (2) DJB improves glucose control in obese mice, which is mainly dependent on the decrease in body weight; (3) MORs and by extension dietary protein sensing are likely not involved in the beneficial outcomes induced by DJB. Therefore, this work shows that DJB surgery in mice is a suitable model to decipher the mechanisms underlying the rapid T2D improvement after RYGB, since DJB is less difficult to realize than RYGB and promotes the same beneficial effects on body weight and glucose homeostasis. Since benefits induced by bariatric surgery seem to be the result of a combination of several complex mechanisms, studies from various mouse models (with or without diabetes, obese or lean) combined with pair-feeding/weight-matching approaches should be useful to improve our knowledge in the mechanisms underlying gastric bypass surgeries.

## Additional Information

**How to cite this article**: Barataud, A. *et al*. Absence of Role of Dietary Protein Sensing in the Metabolic Benefits of Duodenal-Jejunal Bypass in the Mouse. *Sci. Rep.* **7**, 44856; doi: 10.1038/srep44856 (2017).

**Publisher's note:** Springer Nature remains neutral with regard to jurisdictional claims in published maps and institutional affiliations.

## References

[b1] Rubino F. From bariatric to metabolic surgery: definition of a new discipline and implications for clinical practice. Curr. Atheroscler. Rep. 15, 369 (2013). 24194467 10.1007/s11883-013-0369-x

[b2] Sjöström L. . Lifestyle, diabetes, and cardiovascular risk factors 10 years after bariatric surgery. N. Engl. J. Med. 351, 2683–2693 (2004). 15616203 10.1056/NEJMoa035622

[b3] Pories W. J. . Who would have thought it? An operation proves to be the most effective therapy for adult-onset diabetes mellitus. Ann. Surg. 222, 339–350; discussion 350–352 (1995). 10.1097/00000658-199509000-00011 PMC1234815 7677463

[b4] Adams T. D. . Health outcomes of gastric bypass patients compared to nonsurgical, nonintervened severely obese. Obes. Silver Spring Md 18, 121–130 (2010). 10.1038/oby.2009.178 PMC2864142 19498344

[b5] Brethauer S. A. . Can diabetes be surgically cured? Long-term metabolic effects of bariatric surgery in obese patients with type 2 diabetes mellitus. Ann. Surg 258, 628–636; discussion 636–637 (2013). 10.1097/SLA.0b013e3182a5034b PMC4110959 24018646

[b6] Schauer P. R. . Effect of laparoscopic Roux-en Y gastric bypass on type 2 diabetes mellitus. Ann. Surg. 238, 467–484; discussion 84–85 (2003). 14530719 10.1097/01.sla.0000089851.41115.1b PMC1360104

[b7] Weigle D. S. . A high-protein diet induces sustained reductions in appetite, ad libitum caloric intake, and body weight despite compensatory changes in diurnal plasma leptin and ghrelin concentrations. Am. J. Clin. Nutr. 82, 41–48 (2005). 16002798 10.1093/ajcn.82.1.41

[b8] Veldhorst M. . Protein-induced satiety: effects and mechanisms of different proteins. Physiol. Behav. 94, 300–307 (2008). 18282589 10.1016/j.physbeh.2008.01.003

[b9] Jakubowicz D., Froy O., Wainstein J. & Boaz M. Meal timing and composition influence ghrelin levels, appetite scores and weight loss maintenance in overweight and obese adults. Steroids 77, 323–331 (2012). 22178258 10.1016/j.steroids.2011.12.006

[b10] Aller E. E. J. G. . Weight loss maintenance in overweight subjects on ad libitum diets with high or low protein content and glycemic index: the DIOGENES trial 12-month results. Int. J. Obes. 2005, doi: 10.1038/ijo.2014.52 (2014). 24675714

[b11] Claessens M., van Baak M. A., Monsheimer S. & Saris W. H. M. The effect of a low-fat, high-protein or high-carbohydrate ad libitum diet on weight loss maintenance and metabolic risk factors. Int. J. Obes. 2005 33, 296–304 (2009). 10.1038/ijo.2008.278 19153580

[b12] Gannon M. C., Nuttall F. Q., Saeed A., Jordan K. & Hoover H. An increase in dietary protein improves the blood glucose response in persons with type 2 diabetes. Am. J. Clin. Nutr. 78, 734–741 (2003). 14522731 10.1093/ajcn/78.4.734

[b13] Nuttall F. Q. & Gannon M. C. Metabolic response of people with type 2 diabetes to a high protein diet. Nutr. Metab. 1, 6 (2004). 10.1186/1743-7075-1-6 PMC524031 15507157

[b14] Duraffourd C. . Mu-opioid receptors and dietary protein stimulate a gut-brain neural circuitry limiting food intake. Cell 150, 377–388 (2012). 22771138 10.1016/j.cell.2012.05.039

[b15] Fickel J., Bagnol D., Watson S. J. & Akil H. Opioid receptor expression in the rat gastrointestinal tract: a quantitative study with comparison to the brain. Brain Res. Mol. Brain Res. 46, 1–8 (1997). 9191072 10.1016/s0169-328x(96)00266-5

[b16] Bagnol D., Mansour A., Akil H. & Watson S. J. Cellular localization and distribution of the cloned mu and kappa opioid receptors in rat gastrointestinal tract. Neuroscience 81, 579–591 (1997). 9300443 10.1016/s0306-4522(97)00227-3

[b17] Sternini C., Patierno S., Selmer I.-S. & Kirchgessner A. The opioid system in the gastrointestinal tract. Neurogastroenterol. Motil. Off. J. Eur. Gastrointest. Motil. Soc. 16 Suppl 2, 3–16 (2004). 10.1111/j.1743-3150.2004.00553.x 15357847

[b18] Matthes H. W. . Loss of morphine-induced analgesia, reward effect and withdrawal symptoms in mice lacking the mu-opioid-receptor gene. Nature 383, 819–823 (1996). 8893006 10.1038/383819a0

[b19] Liu W. . Establishment of duodenojejunal bypass surgery in mice: a model designed for diabetic research. Microsurgery 28, 197–202 (2008). 18286660 10.1002/micr.20454

[b20] Woods M. . Antidiabetic effects of duodenojejunal bypass in an experimental model of diabetes induced by a high-fat diet. Br. J. Surg. 98, 686–696 (2011). 21381002 10.1002/bjs.7400

[b21] Nestoridi E., Kvas S., Kucharczyk J. & Stylopoulos N. Resting energy expenditure and energetic cost of feeding are augmented after Roux-en-Y gastric bypass in obese mice. Endocrinology 153, 2234–2244 (2012). 22416083 10.1210/en.2011-2041

[b22] Kucharczyk J., Nestoridi E., Kvas S., Andrews R. & Stylopoulos N. Probing the mechanisms of the metabolic effects of weight loss surgery in humans using a novel mouse model system. J. Surg. Res. 179, e91–98 (2013). 22504136 10.1016/j.jss.2012.02.036

[b23] Hao Z., Zhao Z., Berthoud H.-R. & Ye J. Development and verification of a mouse model for Roux-en-Y gastric bypass surgery with a small gastric pouch. PloS One 8, e52922 (2013). 23326365 10.1371/journal.pone.0052922 PMC3543411

[b24] Mokadem M., Zechner J. F., Margolskee R. F., Drucker D. J. & Aguirre V. Effects of Roux-en-Y gastric bypass on energy and glucose homeostasis are preserved in two mouse models of functional glucagon-like peptide-1 deficiency. Mol. Metab. 3, 191–201 (2014). 24634822 10.1016/j.molmet.2013.11.010 PMC3953682

[b25] Lan Z. . Development of techniques for gastrojejunal bypass surgery in obese mice. Microsurgery 30, 289–295 (2010). 20049916 10.1002/micr.20746

[b26] Ullrich J., Ernst B., Wilms B., Thurnheer M. & Schultes B. Roux-en Y gastric bypass surgery reduces hedonic hunger and improves dietary habits in severely obese subjects. Obes. Surg. 23, 50–55 (2013). 22941334 10.1007/s11695-012-0754-5

[b27] Laurenius A. . Decreased energy density and changes in food selection following Roux-en-Y gastric bypass. Eur. J. Clin. Nutr. 67, 168–173 (2013). 23299713 10.1038/ejcn.2012.208

[b28] Kovatcheva-Datchary P. . Dietary Fiber-Induced Improvement in Glucose Metabolism Is Associated with Increased Abundance of Prevotella. Cell Metab. 22, 971–982 (2015). 26552345 10.1016/j.cmet.2015.10.001

[b29] De Vadder F. . Microbiota-Produced Succinate Improves Glucose Homeostasis via Intestinal Gluconeogenesis. Cell Metab. 24, 151–157 (2016). 27411015 10.1016/j.cmet.2016.06.013

[b30] Everard A. . Cross-talk between Akkermansia muciniphila and intestinal epithelium controls diet-induced obesity. Proc. Natl. Acad. Sci. USA 110, 9066–9071 (2013). 23671105 10.1073/pnas.1219451110 PMC3670398

[b31] Palleja A. . Roux-en-Y gastric bypass surgery of morbidly obese patients induces swift and persistent changes of the individual gut microbiota. Genome Med. 8, 67 (2016). 27306058 10.1186/s13073-016-0312-1 PMC4908688

[b32] Zhang H. . Human gut microbiota in obesity and after gastric bypass. Proc. Natl. Acad. Sci. USA 106, 2365–2370 (2009). 19164560 10.1073/pnas.0812600106 PMC2629490

[b33] Plovier H. . A purified membrane protein from Akkermansia muciniphila or the pasteurized bacterium improves metabolism in obese and diabetic mice. Nat. Med., doi: 10.1038/nm.4236 (2016). 27892954

[b34] Liou A. P. . Conserved shifts in the gut microbiota due to gastric bypass reduce host weight and adiposity. Sci. Transl. Med. 5, 178ra41 (2013). 10.1126/scitranslmed.3005687 PMC3652229 23536013

[b35] Zechner J. F. . Weight-independent effects of roux-en-Y gastric bypass on glucose homeostasis via melanocortin-4 receptors in mice and humans. Gastroenterology 144, 580–590.e7 (2013). 23159449 10.1053/j.gastro.2012.11.022 PMC3835150

[b36] Tabarin A. . Resistance to diet-induced obesity in mu-opioid receptor-deficient mice: evidence for a ‘thrifty gene’. Diabetes 54, 3510–3516 (2005). 16306369 10.2337/diabetes.54.12.3510

[b37] Zuberi A. R., Townsend L., Patterson L., Zheng H. & Berthoud H.-R. Increased adiposity on normal diet, but decreased susceptibility to diet-induced obesity in mu-opioid receptor-deficient mice. Eur. J. Pharmacol. 585, 14–23 (2008). 18396272 10.1016/j.ejphar.2008.01.047 PMC2430069

[b38] Yan S. . Reduction of intestinal electrogenic glucose absorption after duodenojejunal bypass in a mouse model. Obes. Surg. 23, 1361–1369 (2013). 23585076 10.1007/s11695-013-0954-7

[b39] Pillot B., Soty M., Gautier-Stein A., Zitoun C. & Mithieux G. Protein feeding promotes redistribution of endogenous glucose production to the kidney and potentiates its suppression by insulin. Endocrinology 150, 616–24 (2009). 18845639 10.1210/en.2008-0601

[b40] Swenson B. R. . The effect of a low-carbohydrate, high-protein diet on post laparoscopic gastric bypass weight loss: a prospective randomized trial. J. Surg. Res. 142, 308–313 (2007). 17631904 10.1016/j.jss.2007.02.052

[b41] Faria S. L., de Oliveira Kelly E., Lins R. D. & Faria O. P. Nutritional management of weight regain after bariatric surgery. Obes. Surg. 20, 135–139 (2010). 18575942 10.1007/s11695-008-9610-z

[b42] Moizé V. . Protein intake and lean tissue mass retention following bariatric surgery. Clin. Nutr. Edinb. Scotl. 32, 550–555 (2013). 10.1016/j.clnu.2012.11.007 23200926

